# Discordance between clinical and immunological ART eligibility criteria for children in Malawi

**DOI:** 10.1186/1756-0500-7-666

**Published:** 2014-09-22

**Authors:** Bernadette O’Hare, Danny A Milner, Laura Newberry, Isaac Pelani, Ken Malisita

**Affiliations:** University of St Andrews and College of Medicine, Private Bag 360, Chichiri, Blantyre Malawi; Pathology, Harvard Medical School, Boston, MA USA; University of Malawi College of Medicine, Blantyre, Malawi; Department of Paediatrics, Queen Elizabeth Central Hospital, Blantyre, Malawi; Queen Elizabeth Central Hospital, Blantyre, Malawi

**Keywords:** HIV treatment, Pediatrics, Africa, Malawi, CD4 count, WHO clinical staging

## Abstract

**Background:**

Since May 2014, all HIV positive children aged less than five years in Malawi are eligible for ART. For children older than five years they are eligible if they are in WHO stage III/IV, if stage I/II, if their CD4 < 500 cells/mm^3^. Our goal was to compare the WHO clinical classification criteria (WHO stage + CD4/age) to CD4 count (CD4/age) on all children. Prior to 2014, children aged 2–5 years in stage I and II were eligible for ART if their CD4 was < 750 cells/mm^3^. We were interested in the increase in numbers of children in this age group who now meet the eligibility criteria and their average CD4 count.

**Methods:**

Data including age, stage and CD4 count were used. We examined the effect of using two different criteria; WHO staging and checking CD4 count if stage I or II versus CD4 count on all, on the numbers of children eligibility for ART in a cohort of 969 children aged 0 to 14 years in Blantyre, Malawi.

**Results:**

Using WHO stage + CD4/age, 786 patients out of 969 would have been treated and 183 would not. Using CD4/age, 745 patients out of 969 would have been treated and 224 would not. Within the 224 patients not treated by CD4 classification, 41 were clinical stage III or IV. The most common staging condition in these 41 children was low weight for age (i.e. underweight). 41% of children age2-5 years have a CD4 count >750.

**Conclusion:**

Most children are correctly started on treatment using recent guidelines. 41% more children <5 years will be started on ART.

## Background

There are 35 million people living with Human Immunodeficiency Virus (HIV) in the world, 25 million of these live in Sub Saharan Africa (SSA) and about 10% are children [1]. In the last decade the expansion of antiretroviral treatment (ART) in resource limited settings has resulted in eight million people receiving ART, and there were 39% fewer deaths from HIV in 2013 in SSA than in 2005 [1]. However there are discrepancies between adults and children in terms of treatment coverage. It is estimated that 28% of children aged less than 15 who required ART were receiving it compared with 57% of adults [2]. To facilitate the scale up of ART the World health Organization (WHO) have published sequential ART guidelines for a public health approach, the most recent being in 2013 [3]. ART eligibility in children relies on both clinical and immunological criteria with the aim to start those who require ART with a minimum delay.

A meta-analysis of untreated HIV infected children in resource poor settings, showed that CD4 count was the strongest predictor of mortality followed by weight for age [4]. It is desirable that all children are immunologically staged at diagnosis by measuring CD4+ T cells using flow cytometry and have sequential tests every three to six months, depending on their proximity to the thresholds for ART initiation, to monitor disease progression [5]. However, in many resource-limited settings, lack of widely available diagnostic facilities to determine CD4 counts means that clinical staging is often the standard of care [6]. The WHO HIV clinical staging classify subjects into one of four categories, stage I to IV, progressing from primary HIV infection to advanced HIV/AIDS. Initiation of ART is recommended if the patient is clinically staged as WHO clinical stage III or IV (WHO, 2007). A CD4 measurement is recommended if the patient is staged as I or II and eligibility is determined if the CD4 is below an age adjusted threshold, see Table 1 for the evolution of recommendations over time [3, 7, 8].

**Table 1:** **Malawian age adjusted thresholds for ART eligibility in children who are in WHO stage I/II**

	WHO 2006/Malawian 2008 guidelines	WHO 2010/Malawian 2011 guidelines	WHO 2013/Malawi 2014
< 12 months	ALL	ALL	ALL
12- 24 months	<20% or <750 cells/mm^3^	ALL	ALL
24-35 months	<20% or <750 cells/mm^3^	<750 cells/mm^3^	ALL
36-59 months	<15% or <350 cells/mm^3^	<750 cells/mm^3^	ALL
>60 months	<15% or <250 cells/mm	<350 cells/mm^3^	<500 cells/mm^3^

Previous studies comparing clinical versus immunologic staging in children have demonstrated that agreement among eligibility criteria is poor. In a study in 2005 in Kinshasa, using WHO 2004 guidelines, 32.6% of children eligible by clinical criteria were not eligible according to immunological criteria (CD4, CD4% or total lymphocyte count) and 34.7% of children eligible on immunological criteria were not eligible by clinical criteria [9]. A discrepancy was also observed in Tanzania, by Johnson et al., who reported that 22% of children who were staged as III/IV did not have severe immunosuppression using WHO 2006 definitions and 52% of children who were staged as stage I/II did have severe immunosuppression [10]. Children who warrant treatment immunologically but who are not eligible using clinical staging alone represent a failure of the staging system. In view of reports of discrepancy, we wished to compare our current practise of determining eligibility by first staging our paediatric HIV antibody positive patients aged > 5 years and only carrying out a CD4 test on those in stage I and II with carrying out a CD4 test on all patients. We wished to evaluate if, by starting children who are staged as III/IV automatically, we were starting children earlier than they needed to be started, in a setting where the durability of ART regimes has not been fully determined and where we have no access to resistance testing in the case of ART failure. We were also interested in the specific categorizations that may inappropriately stage patients due to co-morbidities.

Guidelines on when to initiate ART in children in Malawi have evolved over time in line with WHO guidelines. National guidelines were published in 2008 and the pediatric component of these reflected the WHO 2006 guidelines [7]. These were updated in 2011 [11] and these largely reflected the 2010 WHO guidelines [8]. The Malawian 2014 guidelines [12], based on the WHO 2013 guidelines [3], recommend starting all children aged < 5 years on ART and to use 500 cells/mm^3^ as the threshold for children older than five years. The trend has been towards starting children on ART with higher CD4 counts and towards using absolute numbers of CD4 cells rather than percentage of total lymphocyte counts. Current UK guidelines recommend that children aged < 1 year are universally started on ART but children > 1 year are started on age specific CD4 thresholds [5]. The differences in the recommendations reflect a programmatic approach in countries with limited access to regular laboratory monitoring. We were also interested in the proportion of children who are now eligible by virtue of their age alone and what the average CD4 count of this group is. Our reason for interest in the average CD4 is that hypersensitivity reactions to nevirapine occur in children and are more common in patients with a higher CD4 count [13] and nevirapine is part of our first line regime.

### Objectives

1. In view of the 2014 guidelines, our first objective was to compare doing a clinical stage and carrying out a CD4 test on stage I and II in children >5 years versus the measurement of CD4 count on all patients > 5 years as a means to determine eligibility for ART in children.
2. In view of the recent change of guidelines we wished to look at the average CD4 of children aged 2–5 and the proportion of children in this age group who will automatically be started on ART but who would not have been eligible prior to the WHO 2013/Malawian 2014 guidelines, i.e. the proportion of children aged 2–5 with absolute CD4 counts >750 cells/mm^3^.

## Methods

### Study design

An observational cross sectional study on data routinely collected on all children enrolled for pre ART care between July 2010 and December 2012 was analyzed.

### Study setting

The nurse led pre ART clinic based at the Queen Elizabeth Central Hospital in Blantyre, Malawi. Children are referred to this clinic after their HIV diagnosis has been made from inpatient services and from surrounding urban clinics. HIV testing relies on two rapid tests for HIV-1 and HIV-2, Determine and Unigold (Determine; Abbott Laboratories, Abbott Park, Illinois, USA and Uni-Gold; Trinity Biotech, Bray, Ireland). The function of the clinic is to clinically stage the patients and to perform CD4 count. In September 2011, the Malawian Guidelines recommended that if a patient was staged as stage III or IV, they should not have a CD4 measured but rather start ART immediately. However, prior to this time a baseline CD4 was performed on all patients, therefore we have clinical staging data and CD4 results on patients in all WHO clinical stages. There is no user fee charged for this service and the clinic is open five days each week, morning and afternoon.

### Participants

All Children aged between 0–14 years of age, from July 2010 and December 2012, who were seen in the pre ART at Queen Elizabeth Central Hospital were examined and staged by WHO criteria. Limited data were recorded, but those variables important for staging were noted. WHO clinical staging is routinely performed. The condition which resulted in them being assigned to a given stage was generally but not always recorded. CD4 counts are performed by the central laboratory at QECH using the Beckman Coulter Flow Care CD4 detection kit, and recorded in the database.

### Ethical approval

The study received ethical approval from the College of Medicine ethics committee (COMREC).

### Variables

Name, age and date of birth if known, weight, height, Z score of weight for height, weight for age if < 5 years, clinical stage and CD4 count is collected for all patients attending the pre ART clinic.

### Statistical methods

Using the data from our pre ART clinic, we compared two possible algorithms as a means to determine eligibility for ART, see Table 2. Our practice since May 2014 is to start all children < 5 years on ART, therefore this group was treated the same in both algorithms.

**Table 2:** **CD4 counts on stage I and II versus CD4 counts on all – a comparison of two algorithms**

Algorithm I – WHO stage + CD4/age	Algorithm II - CD4/age
*Age < 5 TREAT*	*AGE < 5 = TREAT*
*Age > 5 Stage III or IV TREAT*	*Age > 5*
*Stage I or II*	*CD4 < 500 = TREAT*
*CD4 < 500 = TREAT*	*CD4 > 500 = Do not Treat*
*CD4 > 500 = Do not Treat*

1. Clinical staging on all children >5 years and carry out a CD4 count on those in stage I and II, we call this WHO stage + CD4/age algorithm or algorithm 1.
2. The measurement of CD4 count on all children > 5 years, we call this cd4/age algorithm or algorithm 2.

We compared using Fisher’s Exact.

## Results

From our clinical database, we had 1591 patients who had been encountered in clinic. Complete data for analysis was available for 969 patients (age, WHO stage, CD4 count). The distribution of patients by age bands which are currently and recently used to determine treatment and WHO clinical stage for the 969 patients in the study is shown in Table 3. The median age in each stage and the median and interquartile range of the CD4 counts in the different WHO stages are summarized in Table 4, as expected the CD4 counts are higher in the lower stages.

**Table 3:** **The distribution of patients by age category and WHO clinical stage**

	WHO stage				
Age	1	2	3	4	Total
< 2	59	12	43	11	125
2-5 yrs	133	29	71	6	239
> 5	326	143	126	10	605
Total	518	184	240	27	969

**Table 4:** **The median [IQR] age (in months) and absolute CD4 count for the four WHO clinical stages**

	WHO stage			
Age	1	2	3	4
Median age and IQR in months in each stage	72(41–108)	84(60–127)	63(27–111)	26(20–96)
< 2	599 [343–1003]	698 [272–1329]	546 [259–995]	389 [233–1037]
2-5 yrs	667 [393–945]	618 [457–884]	678 [359–929]	410 [335–592]
> 5	480 [324–724]	426 [255–597]	332 [108–634]	474 [199–973]

**Figure 1:**
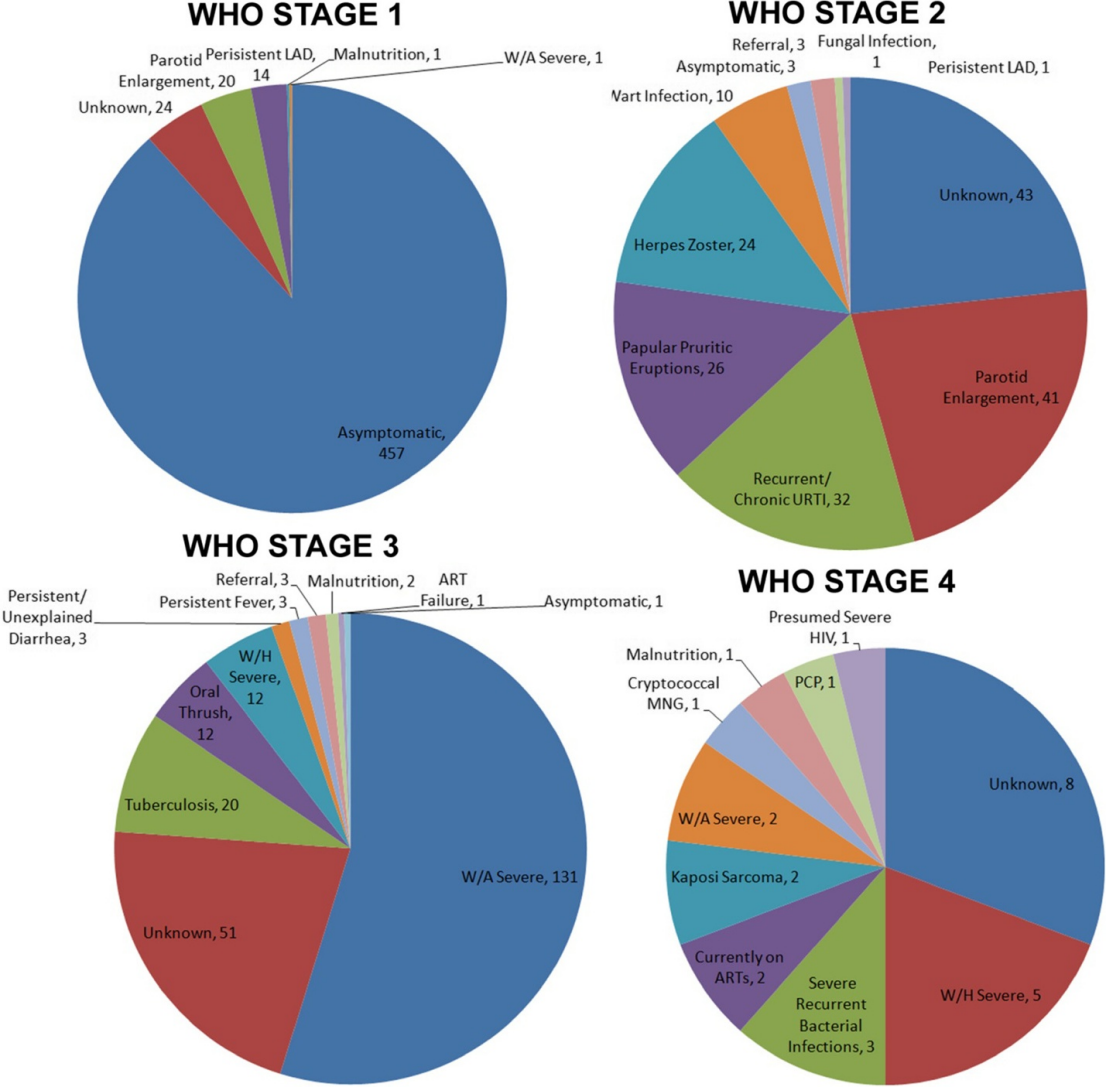
**A summary of the criteria for classifying patients by WHO clinical stage.**

Based on the WHO staging system (algorithm 1, WHO stage + CD4/age), 786 patients out of 969 would have been treated and 183 would not, Table 5. See Figure 1 for a summary of the criteria for classifying patients by WHO clinical stage. Based on the CD4 classification (algorithm 2, CD4/age, ignoring stage), 745 patients out of 969 would have been treated and 224 would not. Within the 224 patients not treated by CD4/age classification, 41 were clinical stage 3 or 4 and would have been treated using the WHO + CD4/age criteria. CD4 counts for these 41 discordantly classified children are summarized in Table 6, with a range of 667–1182 cells/mm^3^. The most common clinical feature was severe weight for age (21 children or 51%), tuberculosis (4 children or 10%), severe recurrent bacterial infections (2 children or 5%) and unknown (6 children or 15%).

**Table 5:** **The number of patients and median absolute CD4 count with IQR are shown by each of the classifications**

	CD4/Age (algorithm 2)		
WHO stage, CD4 count (algorithm 1)	No Treat	Treat	Total algorithm 1
No Treat	183	--	183
Treat	41	745	786
Total algorithm 2	224	745	969

**Table 6:** **The 41 discordant treatment patients by age category and stage with median CD 4 counts and IQR given below**

Stage	
III	IV
36	6
810 [667–1182]	973 [752–992]

The average CD4 of children aged 2–5 years in each of the clinical stages ranged between 667 in stage I to 410 in stage IV, Table 4. The cut off for this age group in the WHO 2010/Malawian 2011 guidelines was 750 cells/mm^3^. There were 239 children in this age group, 97 children had CD4 counts of >750 and 142 had CD4 counts <750, Table 7. 41% of children in this age group would not have been eligible for ART using the WHO 2010/Malawian 2011 guidelines but are eligible using the WHO 2013/Malawian 2014 guidelines.

**Table 7:** **Children aged 2–5 eligible for ART on the WHO 2013/Malawian 2014 guidelines**

Age	<750 cells/mm	>750 cells	Total
2-5 years	142	97	239
%	59%	41%	100%

## Discussion

### Principal findings

Using the WHO 2013 guidelines, more children in our clinic qualify for ART treatment with the WHO + CD4/age classification than the CD4/age classification only. If patients were immunologically classified using CD4 counts and age only, there would have been 41 fewer children eligible for ART. These children were classified as Stage 3 or 4 but have CD4 counts > 500 cells/mm^3^. The most common clinical feature which resulted in children who had relatively high CD4 counts being classified as stage III/IV was weight for age below the 3rd centile i.e. underweight. Although all measures of anthropometric failure are more prevalent in HIV positive children [14], low weight for age is common in all Malawian children; 3- 5% of children aged < 5 years are severely underweight (weight for age is < − 3SD) [15]. This may result in children being assigned to WHO stages III/IV despite a relatively good immune system. However the WHO 2013 guidelines results in the majority of children who would benefit from ART receiving it and only a small proportion who are not immunosuppressed starting earlier than required, mostly due to low weight for age.

Among the concerns of premature initiation of ART include the risk for adverse effects as well as resistance to first-line medications. However, this concern may be unfounded. The risk of adverse effects of ARVs and resistance to first-line medications was relatively low in a retrospective cohort study of 1434 Malawian children. Two percent of patients were on alternative first line treatment due to adverse effects and 1.5% were on second-line regimen due to ART failure after a median period on ART of 1.8 years [16]. Five year survival of children on ART in this setting is 96% [13].

Our findings contrast with findings in the adult literature where more adults qualify for ARV treatment with CD4 classification than with the WHO classification. For example, a study in Uganda demonstrated that 53% of the patients in clinical stage I and II would have been eligible for ART with using CD4 counts < 250 cells/mm^3^ and 49% using CD4 counts < 350 cells/mm^3^[17]. A study from Ghana found 29.5% of patients in stage I and II to have CD4 counts < 200 cells/mm^3^[18]. In Malawian adults it was found that 25% of adults in stage I or II had CD4 counts < 250 cells/mm^3^ and 46% had CD4 counts < 350 cells/mm^3^[19].

41% in the 2–5 year age group would not have been eligible using the WHO 2010/Malawi 2011 guidelines are eligible for ART using the WHO 2013/Malawian 2014 guidelines. There is little clinical evidence to support early initiation of ART in children aged 2–5 years [20] and this is not current practice in the west [5], although it may be justified on a programmatic basis where there is limited access to CD4 counts [3].

### Strengths and weakness of the study

This is a retrospective review of chart data which may contain some errors.

### Strengths and weakness compared to other studies

The sample sizes in this study are larger than other studies and use the most recent guidelines.

The discordant rate is much lower than that reported by others and this is likely because we have used more recent guidelines for the modelling of our data [9, 10]. A weakness compared to other studies is the fact that the data was not collected prospectively [10].

## Conclusions

Our current practise, based on the most recent guidelines, of clinically staging children, immediately starting all children in stage III and IV and doing CD4 counts on children who are found to be in stage I and II is likely to result in the majority of children being correctly started on treatment. Children who are staged as III or IV and who have a high CD4 count are likely to have been staged due to low weight for age. Treatment monitoring needs to take this into account and viral loads in cases of suspected failure should be done when a child on treatment is falling across centiles rather than low weight for age. The absence of a baseline CD4 in children who presented in stage III or IV means that immunological failure may be difficult to diagnose and ideally virological failure should be confirmed using viral load. The WHO 2013/Malawian 2014 guidelines will result in an increase in children aged 2–5 being started on ART and this will need to be monitored in terms of adverse effects as a result of starting children with high CD4s on ART and the known association between high CD4 and hypersensitivity when using nevirapine.
